# Risk of reoperation after TEP, TAPP, and Lichtenstein repair for primary groin hernia: a register-based cohort study across two nations

**DOI:** 10.1007/s10029-025-03374-z

**Published:** 2025-05-30

**Authors:** Kristoffer Andresen, Lovisa Kroon, Henrik Holmberg, Pär Nordin, Jacob Rosenberg, Stina Öberg, Hanna de la Croix

**Affiliations:** 1 Center for Perioperative Optimization, Department of Surgery, Copenhagen University Hospital– Herlev Hospital, Copenhagen, Denmark; 2 Department of Surgery, Institute of Clinical Sciences, Sahlgrenska Academy, University of Gothenburg, Gothenburg, Sweden; 3 Department of Epidemiology and Global Health, Umeå University, Umeå, Sweden; 4 Department of Surgical and Perioperative Sciences, Umeå University, Umeå, Sweden; 5 Department of Surgery, Gothenburg, Sweden; 6 Surgical outpatient clinic, Hallands Hospital Kungsbacka, Sahlgrenska University Hospital/Östra Hospital, Tölövägen 3, 434 40 Sweden

**Keywords:** Hernia, Inguinal hernia, Femoral hernia, Recurrence, Reoperation, TEP, TAPP, Lichtenstein

## Abstract

**Purpose:**

Annually, more than 24,000 groin hernia repairs are performed in Sweden and Denmark, approximately 12,000 of which are laparoscopic like totally extraperitoneal (TEP) and transabdominal preperitoneal (TAPP) repairs. TEP is the preferred technique in Sweden, whereas TAPP is preferred in Denmark. This study aimed to assess the risk of reoperation for recurrence following TAPP, TEP, and Lichtenstein techniques.

**Method:**

Prospectively collected data from the Danish Hernia Database and the Swedish Hernia Register were utilized for this observational register-based study. Primary groin hernia repairs utilizing TEP, TAPP, or Lichtenstein techniques between 2004 and 2020 were included. The primary outcome was the reoperation rate for recurrence analyzed using both crude reoperation rates and Cox proportional hazard regression analysis.

**Results:**

During 17 years, 347,912 primary groin hernia repairs were performed, of which 12% were TEP, 15% TAPP, and 74% Lichtenstein repairs. In males, the risk of reoperation was higher after TEP than after TAPP (HR 1.38, 95% CI 1.27–1.5) and Lichtenstein (HR 1.44, 95% CI 1.36–1.53). In females, Lichtenstein repair had a higher risk than the laparoscopic approaches, with no significant difference between TAPP and TEP.

**Conclusion:**

Our study demonstrated low rates of reoperation for recurrence after Lichtenstein, TEP, and TAPP repairs. In men, TEP repair is associated with an increased risk of reoperation for recurrence compared with Lichtenstein and TAPP repair. For females, the laparoscopic approaches were superior to the Lichtenstein repair. These findings emphasize the importance of international comparative studies to optimize hernia management strategies.

## Introduction

One of the most frequently performed procedures in general surgical practice is groin hernia repair, with an estimated 20 million such procedures performed worldwide each year [1]. The risk of recurrence has decreased since the introduction of mesh reinforcement [2], and alongside the traditional Lichtenstein repair, laparoscopic approaches have gained wide acceptance [3]. In the updated international guidelines for the management of groin hernia, the laparoscopic approach is recommended as first choice for primary groin hernia, provided the surgical clinic has the necessary expertise and equipment [4]. The two main methods of laparoscopic repairs are the transabdominal preperitoneal (TAPP) and totally extraperitoneal (TEP) techniques. TEP repair involves surgery performed entirely in the preperitoneal space, encompassing dissection, overview of the myopectineal orifice, hernia reduction, and mesh placement [5, 6]. Conversely, during a TAPP procedure, the preperitoneal space is accessed via the abdominal cavity with mesh placement following an approach similar to TEP surgery [7–11].

Previous studies have demonstrated comparable outcomes regarding early postoperative pain, chronic pain, wound infection, and hematoma formation for both TAPP and TEP techniques [9, 10, 12–15], but an international consensus is still lacking regarding the optimal laparoscopic surgical approach for primary groin hernia repair. Until now, no register-based study with long-term follow-up and national coverage has been published on the subject. In Sweden and Denmark, approximately 12–13,000 laparoscopic groin hernia procedures are performed annually [16, 17]. Due to historical preferences, TEP repair has been favored by Swedish surgeons, while TAPP repair is the primary choice in Denmark. Sweden and Denmark are neighboring Scandinavian countries both benefiting from nationwide groin hernia databases with national coverage. Combining these databases offers a unique opportunity to study and compare the risk of reoperation for recurrence following primary groin hernia repair using TEP, TAPP, and Lichtenstein techniques in both men and women.

The aim of this study was to assess the risk of reoperation for recurrence following the TAPP, TEP, and Lichtenstein techniques for groin hernia repair.

## Methods

This study was an observational register-based cohort study with prospectively collected data from the Danish Hernia Database and the Swedish Hernia Register, and a protocol for the study has been published [18]. Eligible for the study were all primary groin hernia operations performed as TAPP, TEP, or Lichtenstein on participants 18 years or older, as participants under 18 years are not included in the Danish database. To ensure the data’s comparability, only participants with their primary operation registered between January 2004 and December 2020 were included. Participants were excluded in case of missing or largely incomplete information on hernia anatomy (inguinal, including lateral/medial/pantaloon, or femoral), method of surgery, or if the first repair was registered as a recurrence. Suture reconstruction without mesh for hernia repair was excluded from the study.

The Swedish Hernia Register, established in 1992, is a voluntary register with a reported national coverage of 94% [16, 19]. In comparison, the Danish Hernia Database, founded in 1998, is a mandatory register with a 93% national coverage [17]. Variables in both registers have changed over time, and only parameters included in both databases are used for analysis to ensure comparability. The primary purpose of both databases is to improve surgical outcomes by reporting metrics such as reoperation rates, complications, and for the Swedish register also chronic pain.

In both Denmark and Sweden, all citizens are assigned a unique personal identification number, which facilitates follow-up and registration for reoperation regardless of their location within the country [20]. Thus, these nationwide register studies exhibit high external validity of the data and enable long-term follow-up. Surgeons register data prospectively in association with the surgery, including variables such as patient characteristics, sex, and age, as well as hernia-specific details including defect size, type of hernia (e.g. inguinal, including medial, lateral, or pantaloon), femoral, and combined hernia (inguinal and femoral), and hernia side (left/right/bilateral). Additionally, details on operative procedure such as surgical approach (e.g. TAPP, TEP, Lichtenstein, or other methods), type of mesh, and fixation method (suture, tacks, no mesh fixation) are registered.

Surgery for a primary inguinal hernia was regarded as the exposure variable and reoperation for recurrence was the primary outcome measure. Participants were classified as having undergone a reoperation if two repairs were recorded for the same groin, or the database noted the reoperation as being for recurrence. The unit of analysis was groins, and each groin was analyzed separately for participants undergoing bilateral repair.

### Statistical methods

Characteristics of included participants are presented separately for males and females and classified by method of surgery i.e., TEP, TAPP, or Lichtenstein. Males and females were also analyzed separately, as previously planned^18^. Crude reoperation rates for recurrence were calculated as the number of reoperations divided by the number of participants in each group without considering the follow-up time. The risk of reoperation for recurrence was assessed using Cox proportional hazards regression analyses. Estimates are presented as hazard ratios (HR) with 95% confidence interval (CI), and p *≤* 0.05 was considered statistically significant. Hernia anatomy, participant age, and method of repair were included as covariates in the Cox proportional hazards regression analysis because they were considered potential contributors to recurrence risk based on literature review [2, 21].

Kaplan-Meier plots were used to depict the cumulated reoperation rates, which were compared with a Log Rank test. Participants were censored in case of death, emigration, or end of study period. R version 4.2.2 (2022-10-31) within RStudio 2022.07.1, build 554, with the packages dplyr, tidyverse, survival, ggplot2, and survminer was used for all data handling, statistical analysis, and production of graphs.

### Ethical considerations

In accordance with Danish legislation, ethical approval from an institutional review board is not necessary for accessing data from the Danish hernia database for this study. Authorization to access data from the Swedish hernia register was obtained through approval from the Regional Ethics Board of Gothenburg (Dnr 416 − 17 and 2021 − 00386). Approval was obtained from the Danish Data Protection Agency, approval number: P-2023-63, and from the Danish Regional Quality Assurance Program, approval number: DHDB-2023-01-10. All statistical analyses were performed in Denmark. Data are presented in aggregated form, ensuring the anonymity of individual patients. Participation in the registers does not influence patient treatment.

## Results

Between January 2004 and December 2020, a total of 347,912 primary groin hernia repairs were registered and found eligible for inclusion in the study. Table 1 presents the demographics of the included patients subdivided into males and females. In total, 255,843 (74%) Lichtenstein, 51,857 (15%) TAPP, and 40,212 (12%) TEP procedures were included. Most participants were men, and mean age ranged from 56 to 64 years. Participants undergoing Lichtenstein repair were slightly older compared with the laparoscopic (TEP and TAPP) group and received higher rates of local and regional anesthesia. The follow-up duration varied between the groups, with a median follow-up ranging from 5.3 after TEP to 10.4 years after Lichtenstein. The crude reoperation rates for recurrence were overall low, with less than 4% for all repairs in males but 5.1% following Lichtenstein repair in females (Table 2).

**Table 1:** Demographics of included patients. Regional refers to spinal or epidural Blockade. N: number; SD: standard deviation

Female	TAPP, *n* (%)	TEP, *n* (%)	Lichtenstein, *n* (%)
n	10,142	7,660	10,959
Age, mean (SD)	59.6 (16.8)	57.6 (17.3)	64.3 (17.1)
Unilateral	8,157 (80.4)	5,898 (77.0)	10,737 (98.0)
Anesthesia			
General	10,110 (99.7)	7,634 (99.7)	8,163 (74.5)
Local	14 (0.1)	21 (0.3)	1,891 (17.3)
Regional	18 (0.2)	5 (0.1)	905 (8.3)
Inguinal hernia			
Medial	1,931 (19)	1,048 (14)	3,642 (33)
Lateral	5,035 (50)	4,504 (59)	5,599 (51)
Pantaloon: medial + lateral	209 (2)	168 (2)	477 (4)
Unspecified inguinal	105	0 (0)	82 (1)
Femoral hernia	2,214 (22)	1,246 (16)	794 (7)
Combined hernia			
Pantaloon + femoral	54 (1)	18 (0)	14 (0)
Lateral + femoral	366 (4)	300 (4)	118 (1)
Medial + femoral	135 (1)	100 (1)	98 (1)
Other hernia	59 (1)	276 (4)	107 (1)
No hernia found	22 (0)	0 (0)	24 (0)
Follow-up, years mean (SD)	5.4 (3.4)	5.3 (3.3)	10.4 (4.5)
**Male**	**TAPP**,** n (%)**	**TEP**,** n (%)**	**Lichtenstein**,** n (%)**
n	41,715	32,552	244,884
Age mean (SD)	56.7 (14.2)	56.7 (14.0)	61.6 (14.8)
Unilateral	23,462 (56.2)	12,704 (39)	241,127 (98.4)
Anesthesia			
General anesthesia	41,584 (99.7)	32,428 (99.6)	177,418 (72.5)
Local anesthesia	44 (0.1)	91 (0.3)	53,080 (21.7)
Regional anesthesia	87 (0.2)	32 (0.1)	14,373 (5.9)
Inguinal hernia			
Medial	16,457 (39)	12,806 (39)	85,265 (35)
Lateral	21,276 (51)	15,970 (49)	136,942 (56)
Pantaloon: medial + lateral	2,491 (6)	2,367 (7)	20,190 (8)
Combined hernia			
Pantaloon + femoral	105 (0)	69 (0)	86 (0)
Lateral + femoral	210 (1)	228 (1)	189 (0)
Medial + femoral	194 (0)	179 (1)	204 (0)
Femoral (%)	552 (1)	298 (1)	280 (0)
No hernia found (%)	60 (0)	2 (0)	190 (0)
Other hernia (%)	83 (0)	620 (2)	1,025 (0)
Unspecified inguinal (%)	274 (1)	13 (0)	504 (0)
Follow-up years mean (SD)	5.9 (4.0)	6.4 (4.5)	8.9 (4.8)

**Table 2:** Crude reoperation rates for recurrence, not adjusted for follow-up time

Crude reoperation rate		TAPP	TEP	Lichtenstein
Female	n/total	130/10,142	81/7,660	561/10,959
	%	1.3	1.1	5.1
Male	n/total	1,015/41,715	1214/32,552	7,978/244,884
	%	2.4	3.7	3.3

**Fig. 1:**
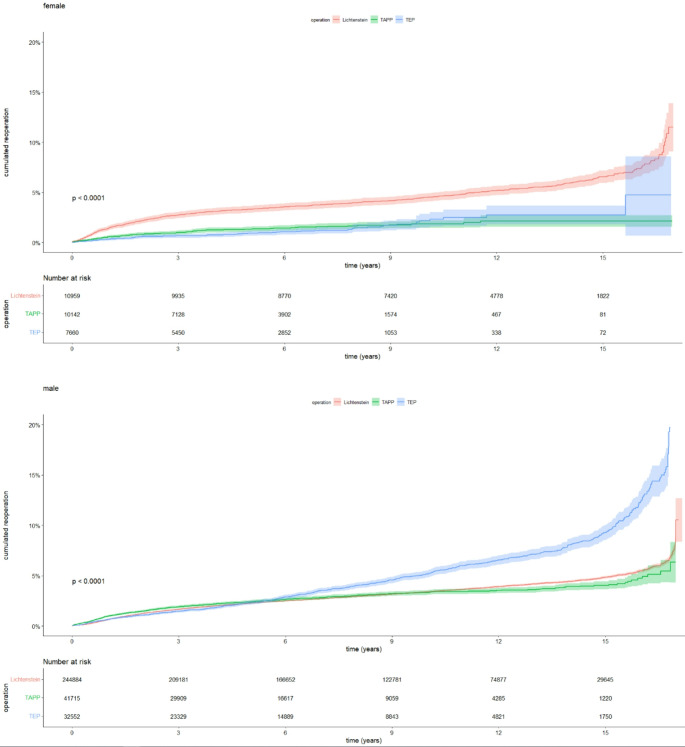
Kaplan-Meier plot illustrating the cumulated reoperation rates for TAPP, TEP, and Lichtenstein procedures. The top plot represents female patients (Log-rank *p* < 0.0001), while the bottom plot represents male patients (Log rank *p* < 0.0001)

Females had a higher proportion of laparoscopic unilateral groin hernia repairs and a greater presence of femoral hernias, whereas males had a higher rate of inguinal hernias, see Table 1. In females, the Kaplan Meier plot showed a higher cumulated reoperation rate for recurrence following Lichtenstein compared with the laparoscopic repairs and overlapping 95% confidence intervals for TAPP and TEP (Fig. 1). For males, there seemed to be no difference in the risk of reoperation for recurrence in the first six years after surgery between the methods, after which the cumulated reoperation rate for recurrence increased more for TEP than the other techniques (Fig. 1).

TAPP and TEP were compared in a Cox proportional hazards regression, demonstrating that there were no differences in the risk of reoperation for recurrence in females, but a higher risk was found in males operated with the TEP technique (Table 3). Both laparoscopic repairs were also compared with the Lichtenstein technique in another Cox proportional hazards regression (Table 4), and Lichtenstein was a risk factor for reoperation in female patients whereas TEP was a risk factor for reoperation for recurrence in male patients. In males, TAPP and Lichtenstein repair carried the same hazard ratio for reoperation for recurrence (Table 4).

**Table 3:** ; Cox regression for the risk of reoperation for recurrence, comparing TAPP and TEP repairs subdivided into female and male patients. HR: hazard ratio; CI: confidence interval; ref: reference; not estimable: some values were not estimable due to the low number of cases

Female	HR	95% CI	*p*	
Age (year)	1	0.99–1.01	0.96	
*Method of repair*				
TAPP	1	ref		
TEP	0.87	0.66–1.15	0.34	
*Anatomy*				
Lateral	1	ref		
Medial	1.7	1.21–2.41	0.003	
Latera + femoral	1.05	0.48–2.26	0.91	
Medial + femoral	1.35	0.43–4.25	0.61	
Femoral	1.38	0.98–1.96	0.07	
Femoral + inguinal (unspecified)	not estimable			
No hernia found	not estimable			
Other hernia	0.38	0.05–2.74	0.34	
Pantaloon + femoral	not estimable			
Pantaloon	1.63	0.71–3.73	0.25	
Inguinal (unspecified)	0.93	0.13–6.71	0.94	
**Male**	**HR**	**95% CI**	**p**	
Age (year)	1.01	1.01–1.01	< 0.005	
*Method of repair*				
TAPP	1	ref		
TEP	1.38	1.27–1.50	< 0.005	
*Anatomy*				
Lateral	1	ref		
Medial	0.77	0.70–0.84	< 0.005	
Lateral + femoral	0.35	0.15–0.84	0.02	
Medial + femoral	0.79	0.44–1.43	0.44	
Femoral	0.57	0.35–0.93	0.025	
Femoral + inguinal (unspecified)	not estimable			
No hernia found	0.87	0.28–2.72	0.82	
Other hernia	0.49	0.26–0.95	0.035	
Pantaloon + femoral	0.36	0.09–1.46	0.15	
Pantaloon	0.84	0.71–0.99	0.044	
Inguinal (unspecified)	1.09	0.52–2.30	0.82	

**Table 4:** ; Cox regression for the risk of reoperation for recurrence, comparing TAPP and TEP with Lichtenstein repair, subdivided into female and male patients. HR: hazard ratio; CI: confidence interval; ref: reference; inguinal (unspecified): medial or lateral hernia

Female	HR	95% CI	*p*
Age	0.99	0.98–0.99	< 0.005
*Method of repair*			
Lichtenstein	1.00		
TAPP	0.41	0.34–0.50	< 0.005
TEP	0.36	0.29–0.47	< 0.005
*Anatomy*			
Lateral	1.00		
Medial	2.54	2.16–2.99	< 0.005
Medial and femoral	2.24	1.22–4.10	0.01
Femoral	1.58	1.23–2.01	< 0.005
Femoral and inguinal unspecified	5.26	0.74–37.42	0.98
Lateral and femoral	1.09	0.58–2.06	0.78
No hernia found	4.32	1.92–9.71	0.02
Other hernia	1.05	0.47–2.36	0.91
Pantaloon and femoral	4.04	0.00 - not estimable	0.98
Pantaloon hernia	1.60	1.08–2.36	0.02
Unspecified inguinal	1.42	0.53–3.80	0.49
**Male**	**HR**	**95% CI**	**p**
Age	1.00	1.00–1.00	0.02
*Method of repair*			
Lichtenstein	1.00	Ref	
TAPP	1.00	0.93–1.06	0.91
TEP	1.44	1.36–1.53	< 0.005
*Anatomy*			
Lateral	1.00	Ref	
Medial	1.66	1.59–1.72	< 0.005
Medial and femoral	1.13	0.70–1.82	0.62
Femoral	1.05	0.73–1.51	0.80
Femoral and inguinal unspecified	3.40	0.48–24.15	0.22
Lateral and femoral	0.87	0.51–1.50	0.62
No hernia found	1.02	0.53–1.96	0.96
Other hernia	0.95	0.68–1.33	0.77
Pantaloon and femoral	1.67	0.87–3.21	0.12
Pantaloon hernia	1.22	1.13–1.32	< 0.005
Unspecified inguinal	2.86	2.10–3.90	< 0.005

**Fig. 2:**
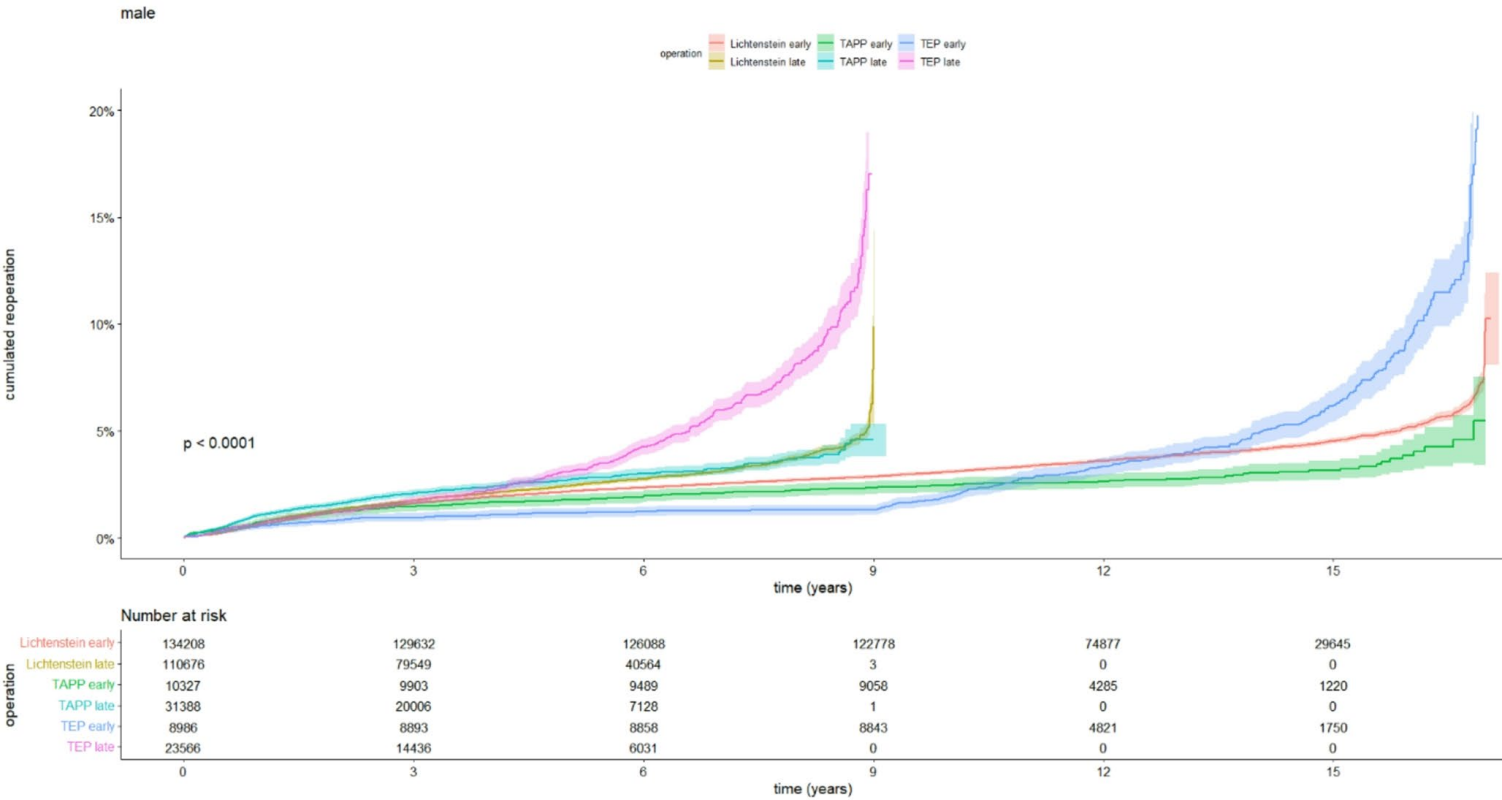
Kaplan-Meier plot illustrating the cumulated reoperation rates for TAPP, TEP, and Lichtenstein procedures, divided into early or late periods (before or after 2012)

To investigate whether the higher risk of reoperation following TEP in male patients was due to a learning curve effect, an exploratory analysis was conducted. The population was divided into an early and a late group depending on the year of surgery being before or after 2012. A Kaplan Meier plot was created showing that for the group of participants operated after 2012, the cumulative risk of reoperation for recurrence was higher, see Fig. 2.

## Discussion

By combining data from nationwide registers in two neighboring countries, we were able to compare the long-term risk of reoperation following more than 300,000 primary groin hernia repairs including 92,069 laparoscopic repairs. This study shows that TAPP was associated with a significantly reduced long-term risk of reoperation for recurrence compared with TEP for men, without a difference for women. While the laparoscopic techniques were superior to the Lichtenstein repair in females, TEP repair was associated with an increased risk of reoperation in men compared with both Lichtenstein and TAPP.

In females, the risk of reoperation was low and did not differ between TAPP and TEP. With a cohort of 28,761 females including 17,802 laparoscopic repairs, any clinically relevant differences would have been found if present. Our study aligns with previous findings indicating that Lichtenstein repair is associated with a higher reoperation rate for recurrence in women [22]. Originally developed as the standard open technique for inguinal hernias, Lichtenstein was not specifically designed for femoral hernias. Notably, previous studies report that femoral hernias were present in 40% of women who underwent reoperation after inguinal hernia repair, suggesting missed femoral hernias [23–25], rather than true recurrences may account for many reoperations. While one might argue that a missed hernia is not a true recurrence, both scenarios indicate a failed repair. Regardless of the underlying cause, clinically relevant recurrences necessitate reoperation and are recorded as such, though the registers do not distinguish between true recurrence and missed hernia. Therefore, to mitigate the risk of recurrence in female patients, we suggest considering a laparoscopic approach whenever feasible, in line with current guidelines [2].

In males, our analysis showed that the reoperation rate for recurrence was overall low for both TAPP and Lichtenstein. However, both crude reoperation rate and hazard ratio indicated a higher risk of reoperation for recurrence after TEP compared with TAPP and Lichtenstein for male patients. To investigate this further, exploratory analysis revealed that TEP repairs demonstrated an elevated risk of reoperation in the latter half of the study period. The estimates at the end of the curve should be interpreted with caution due to the low number of participants at risk. While previous studies have shown no significant difference in intra-abdominal complications between TEP [10, 14] and TAPP, the risk of recurrence remains a critical factor when selecting the type of repair. Notably, the increased hazard ratio for reoperation due to recurrence after TEP has also been highlighted in a previous cohort study based on the Swedish Hernia Register, reporting a hazard ratio of 1.77 with open anterior mesh repair as reference [26].

Lichtenstein repair is a widely recognized tension-free open procedure that has become a cornerstone in hernia surgery. Originally described by Lichtenstein et al. [27], this technique has been refined and adapted over the years to include various national modifications as endorsed through consensus meetings [8, 28, 29]. Renowned for its cost-effectiveness, ease of learning and teaching, and consistent and reproducible outcomes [27, 30], the Lichtenstein repair is a reproducible technique that can be performed under local or regional anesthesia, offering significant versatility.

According to our results, the risk of reoperation for recurrence is lower after TAPP and Lichtenstein compared with TEP repair in males. This observation aligns with findings from the Swedish Hernia Register, demonstrating a higher reoperation rate following laparoscopic repairs, which in Sweden is predominantly TEP repairs [22]. However, several previous studies that report no difference in the risk of reoperation for recurrence are limited by their relatively short follow-up period, typically ranging from 1 to 3 years [10, 11, 31]. Such limited follow-up durations may fail to adequately capture long-term recurrence rates, potentially obscuring meaningful differences between surgical techniques.

Another factor to consider is whether the learning curve for mastering the TEP technique may contribute to the increased risk of recurrence observed in males. For general surgeons, navigating the posterior inguinal area, preperitoneal space, and myopectineal orifices accurately poses a challenge due to the unfamiliarity of this anatomical region. It is worth noting that our study does not differentiate between clinics with varying frequencies of hernia surgery procedures. However, a recent study from the Swedish Hernia Register showed that the risk of reoperation after TEP increased in surgical units performing less than 51 repairs annually [32]. In their study, patients operated at high volume centers had a significantly lower risk of reoperation compared with low-volume counterparts. Consequently, individual centers that perform TEP and TAPP procedures at high volumes may yield excellent results regarding reoperation risk compared with national averages. However, this study shows collective results reflecting routine care in Sweden and Denmark without selection of patients, surgeons, or clinics. Thus, based on the results of this study, one could argue for restricting TEP procedures to high-volume hernia centers where surgeons possess adequate expertise in its execution [33].

In a large part of the world, laparoscopic procedures are not possible to perform due to lack of resources. From this study, we can safely conclude that Lichtenstein repair is a good choice for primary inguinal hernia in men regarding the risk of reoperation for recurrence.

### Strengths and limitations

The strength of this study is the national coverage of the included registers. The absence of patient and surgeon selection bias creates a high external validity that is easily extrapolated to similar surgical settings. The large number of groin hernia repairs included provides a robust dataset for analysis. Given its size, minor data collection errors are likely inconsequential. The data in the two national databases are readily available and registered prospectively without affecting patient treatment.

The data did not distinguish between centers with varying levels of expertise in hernia repair volumes. To mitigate bias, Lichtenstein hernia repair, a procedure performed in both countries, serves as the baseline for comparison.

One limitation of the study is that the true rate of recurrence cannot be accurately measured through the current analysis. It is noteworthy that patients who do not undergo surgical intervention for their recurrence may not be considered to have clinically relevant recurrences. Additionally, the analysis does not encompass aspects related to quality of life, including chronic pain and other factors that significantly influence patient-perceived outcomes following surgery, although these factors are equally important to consider. Another pertinent limitation to acknowledge is the temporal variability in the variables entered into the registers. These variables were not exclusively tailored for the current study’s objectives, and as such, their composition and scope have changed over the study period [34].

## Conclusion

Our study shows that Lichtenstein, TEP, and TAPP repairs demonstrated low rates of reoperation for recurrence. In males, the TEP repair was associated with an increased risk of reoperation for recurrence compared with Lichtenstein and TAPP repair. For females, the laparoscopic approaches were superior to the Lichtenstein repair.
